# Carbon Gain Limitation Is the Primary Mechanism for the Elevational Distribution Limit of *Myriophyllum* in the High-Altitude Plateau

**DOI:** 10.3389/fpls.2018.01129

**Published:** 2018-08-02

**Authors:** Dong Xie, Zhigang Wu, Han Y. H. Chen, Zhong Wang, Qiang Wang, Dan Yu

**Affiliations:** ^1^The National Field Station of Freshwater Ecosystem in Liangzi Lake, College of Life Sciences, Wuhan University, Wuhan, China; ^2^Co-Innovation Center for Sustainable Forestry in Southern China, Nanjing Forestry University, Nanjing, China; ^3^Faculty of Natural Resources Management, Lakehead University, Thunder Bay, ON, Canada

**Keywords:** alpine submerged macrophytes, growth-limitation hypothesis, high-altitude plateau, low temperature, *Myriophyllum*, non-structural carbohydrates

## Abstract

Temperature comprises a major driver for species distribution and physiological processes in alpine plants. For all terrestrial plant species tested to date, elevation associated decreases in temperature have been observed to influence the balance between carbon acquisition and usage; restricting the upper limit of most alpine trees (i.e., treeline). However, such a carbon source-sink balance has not been tested in any alpine aquatic plants, which is an important component of the alpine aquatic ecosystem. The *Myriophyllum* species inhabits a broad range of habitats across the high-altitude plateau. Three *Myriophyllum* species (*Myriophyllum spicatum*, *Myriophyllum verticillatum*, and *Myriophyllum sibiricum*) from 12 water bodies at elevational gradients between 2766 and 5111 m were collected in the Qinghai-Tibetan Plateau. The late growing seasonal concentrations of non-structural carbohydrates (NSC) in the leaves were measured to find how high-altitude conditions influence the carbon balance in aquatic plants. Regression tree analysis separated the 12 water bodies into two groups according to water turbidity (seven water bodies with high turbidity and five water bodies with low turbidity). Overall, leaf NSC concentrations (primarily starch) decreased significantly with increasing elevation in widely distributed *M. spicatum* and *M. verticillatum*. Regression tree analysis indicated that water turbidity (i.e., shady environment) was a strong determinant of leaf NSC. In the low turbidity group (<3.5 NTU), leaf NSC concentrations decreased with increasing elevation; however, in the high turbidity group (>3.5 NTU), leaf NSC concentrations were low and had no association with elevation. Unlike most recent studies in tree species, which show low temperatures limited growth at high-elevations, our results demonstrated that carbon gain limitation is the primary mechanism for the elevational distribution limit of *Myriophyllum* species in the Qinghai-Tibetan Plateau. Moreover, water turbidity moderated the effects of low temperature by masking the expected carbon limitation trend. Therefore, at least two environmental factors (i.e., temperature and light availability) induced photosynthesis decreases might explain the NSC responses for aquatic plants in response to elevation.

## Introduction

At high-elevations, plants often face severe environmental conditions, such as low temperature, ice/snow cover and high ultraviolet-B radiation, which restrict growth, reproduction, and many metabolic functions (Lacoul and Freedman, 2006a; Aichner et al., 2010; Loayza-Muro et al., 2013). Several studies on terrestrial plants have demonstrated the existence of low temperature-adapted patterns in phenology (Potvin, 1986), growth (Shi et al., 2008) and morphology (Alvarez-Uria and Körner, 2007). Although the aquatic environment may affect elevation gradient responses by buffering temperature fluctuations, the diversity of aquatic plant species have been associated with (water) temperature across elevation gradients, ranging from 77 to 4980 m in the Himalayas (Lacoul and Freedman, 2006b). Jones et al. (2003) also revealed that species richness declined with the increase of elevation in Cumbria, United Kingdom (elevation gradients range from 2 to 837 m). These findings are similar to those for terrestrial plants, where previous studies have attempted to utilize environmental factors, such as temperature and water availability, to explain their physiological mechanisms in response to high elevations (Körner, 1998; Hoch et al., 2002; Hoch and Körner, 2009; Fajardo et al., 2011); however, we have little knowledge of the elevational responses in aquatic plants.

Common plant species with extensive distribution may perform well under a broad range of environmental conditions (Joshi et al., 2001). Many aquatic plant species are widely dispersed, reproduce asexually, and often possess limited genetic variation (Santamaría, 2002). The successful propagation of aquatic plants under variable environmental conditions is often linked to their phenotypic plasticity. For instance, Ganie et al. (2014) observed that morphogenic plasticity caused the successful propagation of 10 *Potamogeton* species across habitats with different water flow types in the Kashmir Himalayas. Environmentally induced phenotypic plasticity may lead to rapid changes in plant phenotypic characteristics, which support the survival, reproduction, and dispersal of aquatic plant species across a broad range of habitats (Ganie et al., 2014).

Elevation associated temperature is regarded as one of the major drivers of plant distribution and individual physiological processes, not only for terrestrial plants (Hoch and Körner, 2009), but also for aquatic plants (Rooney and Kalff, 2000). Aquatic plants (particularly submerged macrophytes) are believed to be eurythermic and able to thrive under a wide range of temperatures (Madsen and Brix, 1997). A research from global dataset revealed that physiological acclimation of plants will lead increase of leaf nitrogen (N) and phosphorus (P) concentrations to offset the depressed biochemical efficiencies (e.g., N-rich enzymes and P-rich RNA) in colder, rather than warmer, climates (Reich and Oleksyn, 2004). The changes of leaf N and P concentrations will also regulate carbon (C) acquisition and use in plants (Reich and Oleksyn, 2004). When exposed to low temperatures/cold stress environments, perennial aquatic plant species typically exhibit physiological plasticity, in terms of photosynthesis, storage accumulation and nutrient elements absorption (Madsen and Brix, 1997; Klimeš et al., 1999;Olesen and Madsen, 2000). For instance, Wang et al. (2015) found increased concentrations of leaf N and P in aquatic plants in response to low temperatures in the Qinghai-Tibetan Plateau. Being different from plants in terrestrial habitats, aquatic plants in high elevational water bodies are not only subjected to extreme low-temperature environmental conditions, but also shade stresses (e.g., from high suspended organic and/or inorganic particle concentrations and filamentous algae) (Jackbsen, 2008). Although alpine water bodies are typically clear, some high mountain lakes show specific turbid stages related to the thermal budget of the lakes with cold turbid water inflow (Root et al., 2006). Even within very short distances, some streams may also exhibit very different mean levels and ranges of suspended solids (Jackbsen, 2008). Surprisingly, despite the relatively extensive literature dealing with aquatic plants in response to temperature or shade gradients, to our knowledge, few studies have considered the potentially simultaneous influences of temperature decreases and turbid stages on the distribution of submerged macrophytes at high elevations.

For both terrestrial and aquatic plants, non-structural carbohydrates (NSC) including soluble sugars and starches are common storage molecules, which serve to increase plant survival and recovery in habitats with frequent disturbances (Puijalon et al., 2008; Huber et al., 2012; Adams et al., 2013). Previous studies with terrestrial plants have shown that elevation-induced low temperatures trigger the increased storage of NSC in woody tissue, and growth restriction of multiple tree species in the treeline ecotone (Hoch et al., 2002; Shi et al., 2008; Fajardo et al., 2012). These results indicated that tree cell and tissue formation are initially limited by elevation associated decreases in temperature (i.e., growth-limitation hypothesis, GLH) (Körner, 1998), and not what was previously thought; that low temperatures limited photosynthetic decline, which limited plant growth at high-elevations (i.e., carbon-limitation hypothesis, CLH). A growing number of studies support the GLH in trees (e.g., Shi et al., 2008; Fajardo et al., 2012; Palacio et al., 2014; Hoch, 2015); however, several studies suggested that a direct connection between NSC accumulation and restrained growth was inconclusive (Wiley and Helliker, 2012; Fajardo and Piper, 2014). The most straightforward approach for the resolution of such a debate was the comparison of NSC concentrations of plant species along an elevational gradient (Fajardo et al., 2011). There has only been a single study that measured NSC concentrations in the rhizomes of emergent macrophyte *Phragmites australis* (Cav.) Trin. ex Steud. at two elevations (400 and 1350 m, respectively), and found that the NSC concentrations of rhizomes were higher in the high, rather than the low, elevation site (1350 m, 35.2% compared with 30.5%, respectively) (Klimeš et al., 1999). Hence, a better understanding is required in terms of how the energy storage of aquatic plants responds to a wide range of elevations.

**FIGURE 1:**
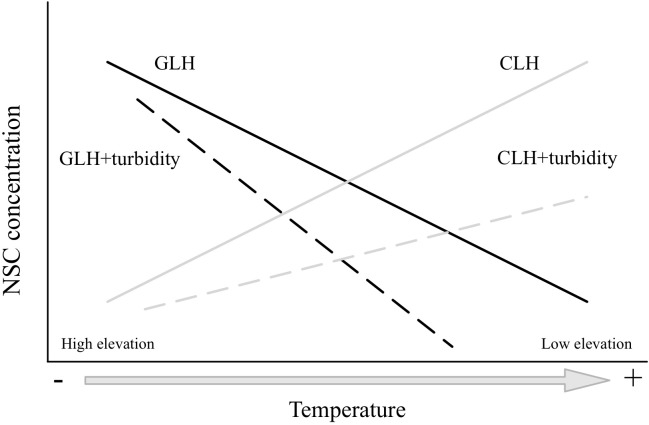
Expected trends of non-structural carbohydrate concentrations (NSC) with elevational gradients (temperature effects) according to the growth-limitation hypothesis (GLH; black lines) and carbon-limitation hypothesis (CLH; gray lines), which considers the alternative effects of water turbidity. The effect of temperature on either the photosynthesis (CLH), or the growth (GLH) is displayed by continuous lines. The alternative impact of water turbidity is displayed by long-dashed lines. If supporting the GLH, a steeper NSC-elevation slope is predicted (black long-dashed line). In contrast, if supporting the CLH, higher NSC concentrations are anticipated to occur at lower elevations (with higher temperatures) (gray long-dashed line) (see text for further details on the expectations).

The principal aim of this study was to test the C acquisition-demand balance in submerged alpine macrophytes in response to variable elevational temperature gradients, and the influence of water turbidity on the response of the C balance to elevation (**Figure 1**). We addressed the following questions: (1) do NSC concentrations of *Myriophyllum* species increase or decrease with elevation; and (2) besides temperature, how do water turbidity alter the C balance in plants in response to an elevational gradient? The genus *Myriophyllum* species were used because these species comprise submerged macrophytes that occupy an extensive range of habitats globally, including high-elevation regions (Cook, 1990; Jackbsen, 2008). The Qinghai-Tibetan Plateau, in China is a unique geographic unit that is subject to harsh environmental conditions (e.g., mean altitude over 4000 m; the average warmest month temperature is below 10°C in large areas). There are multiple shallow lakes, which are covered with aquatic macrophytes during the short growing season (5–6 months). This region provides an ideal platform for the investigation of the responses of plants along an elevational gradient. The genus *Myriophyllum*, primarily *Myriophyllum spicatum*, *Myriophyllum verticillatum*, and *Myriophyllum sibiricum*, represents one of the largest aquatic genera, and inhabits a broad range of habitats across the Qinghai-Tibetan Plateau (Aichner et al., 2010; Wang et al., 2015; Wu et al., 2016). Based on previous trends of NSC concentrations (in trees) with elevational gradients, we anticipated that if increasing elevation (decreased temperatures) reduced plant growth (C consumption) more than photosynthesis (C accumulation), the NSC will increase with elevation (acquisition > demand), supporting the GLH (**Figure 1**; decreasing continuous black line). Conversely, if photosynthesis becomes more limited than growth, in correspondence with increasing elevation, the NSC will decrease (acquisition < demand), which is supportive to the CLH (**Figure 1**; increasing continuous gray line). Moreover, water turbidity may limit C gains in submerged macrophytes (e.g., Lacoul and Freedman, 2006a; Puijalon et al., 2008; Huber et al., 2012); therefore, lower NSC concentrations may be associated with higher water turbidity (**Figure 1**, long-dashed lines). In addition, if temperature acts to limit plant more so than photosynthesis (GLH), a steeper NSC-elevation slope may be predicted, as water turbidity reduces the amount of light that is available for photosynthesis (**Figure 1**, black long-dashed line). In contrast, if temperature limits photosynthesis more than growth (CLH), a steeper NSC-elevation slope is also expected to occur in the opposite direction (**Figure 1**, gray long-dashed line), in that photosynthesis increases with temperature.

## Materials and Methods

### Study Sites

In this study, we sampled 12 water bodies in the Qinghai-Tibetan Plateau, China, spanning a gradient of 1100 km, from east to west. The total elevation gradient spanned from between 2766 and 5111 m. As NSC levels are more stable at the end of the growing season (Madsen, 1997; Hoch and Körner, 2009), all samples were collected during July to August, 2012. The water bodies that we selected to study for *Myriophyllum* species were based on extensive field reconnaissance. We focused on near-pristine (no point sources of pollution, no obvious signs of human impact or grazing) water bodies. More information in regard to the investigation of these water bodies is provided in Supplementary Table 1.

### Field Sampling and Environmental Variables

At each water body, sampling points were established along a sampling line (200 m) every 30 m, where six individual plants (without leaf damage) were selected for sampling. From each sampled individual, upper shoot leaf tissues were collected from between 10:00 and 15:00 h. All plant samples were rinsed/cleaned with tap water, and then bagged, labeled, and sealed over silica gel in order to dry the samples.

To gain a better mechanistic understanding of the variation of NSC, we measured a number of physical characteristics, including conductivity (μS cm^-1^), pH, salinity (%), total dissolved solids (ml l^-1^), dissolved oxygen (ml l^-1^), and turbidity (Nephelometric Turbidity Units, NTU) with a handheld multi-parameter meter (Proplus, YSI, Yellow Springs, OH, United States) at each sampling water body, from 10:00 to 15:00 h. At each designated water body, three water samples were extracted from a depth of 20 cm and initially filtered with a GF/F filter, and then employed to determine water chemical characteristics, including total nitrogen (TN, ml l^-1^), total phosphorus (TP, ml l^-1^), NH_4_^+^ (ml l^-1^), and NO_3_^-^ (ml l^-1^), using an ion chromatography system (ICS-1000, Dionex, Sunnyvale, CA, United States). We derived the growth season temperature (GST) of each water body by entering their geographic coordinates into equations from data collected at meteorological stations across China between the years of 1949 and 1999 (Fang et al., 2001; Wang et al., 2015). The GST is negatively correlated to the elevation (Supplementary Figure 1).

### Non-structural Carbohydrate Analysis

Prior to analysis, all leaf samples were dried to a constant weight at 80°C for 48 h and then ground into a fine powder. The NSC concentrations, including free low molecular weight soluble sugars (SS, including glucose, fructose, and sucrose) and starch, were analyzed using an Agilent 1290/6460 liquid chromatography system and tandem mass spectrometer (Agilent Technologies, Santa Clara, CA, United States) with a Waters XBridge^TM^ BEH Amide 2.5 um 2.1 × 50 mm XP column (Waters, Milford, MA, United States). Approximately 20 mg of plant powder samples were extracted with 2 ml of ethanol (80%, v/v) at 80°C for 30 min., and then centrifuged (10,000 *g* for 10 min.). Subsequent to three extraction processes, the supernatant was utilized for the determination of SS, and the residue was dried with nitrogen for 24 h to dislodge any ethanol. The starch was initially hydrolyzed with diastase (Tokyo Chemical Industry, Japan) (60°C for 10 min.) and then analyzed using the same method as for the determination of SS. We added SS and starch concentrations to obtain NSC concentrations. All sugar and starch concentrations in these tissue samples were expressed as per unit of weight (mg g^-1^).

### Statistical Analysis

To describe and quantify the environment of the individual water bodies, we used the nine characteristic variables (GST, pH, salinity, dissolved oxygen, turbidity, TN, TP, NH_4_^+^, NO_3_^-^) to create principal component analysis (PCA) that together explain nearly 87.35% of the variation present in the original nine variables (Supplementary Figure 2). The resulting components represent two combinations of the original environmental data, with the first one (Comp.1, 54.29%) mainly representing GST, dissolved oxygen, turbidity and total nitrogen, the second one (Comp.2, 33.06%) mainly representing GST, dissolved oxygen and turbidity. The *princomp* function in R was used for conducting the PCA analysis.

Regression tree analysis is well-suited for data that have complex ecological interactions among environmental variables, which forward selected variables (De’ath and Fabricius, 2000). Therefore, our data was initially divided into two water turbidity groups (high turbidity group vs. low turbidity group) using the regression tree, via the analysis of the untransformed NSC concentration data (high turbidity: > 3.97 NTU, six water bodies; low turbidity: < 3.97 NTU, six water bodies, Supplementary Figure 3). There were four water physical and chemical measurements from PCA1 (GST, dissolved oxygen, turbidity, and total nitrogen) that were employed for the regression tree analysis, which was conducted using the *rpart* and *partykit* packages in R. All of the experimental data were then transformed using log(x) or sqrt(x) functions to meet the assumptions of homogeneity in variance and normality.

To study whether the NSC concentration trends of *Myriophyllum* species increased or decreased with elevation, we used the model II regression to fit a linear relationship between the individual NSC concentration and elevational gradients in the high turbidity, and low turbidity groups, respectively (*lmodel2* R package) (Fajardo et al., 2011). However, in the high turbidity group, we found that the slopes of model II regressions did not differ from 0 (*P* > 0.05). We subsequently employed a linear mixed effect model (lme) to compare the mean values between sites, using the GST and turbidity as fixed factors, and the water bodies as the random factors (*nlme* R package). All statistical analyses were performed with R version 3.2.0^1^.

## Results

**FIGURE 2:**
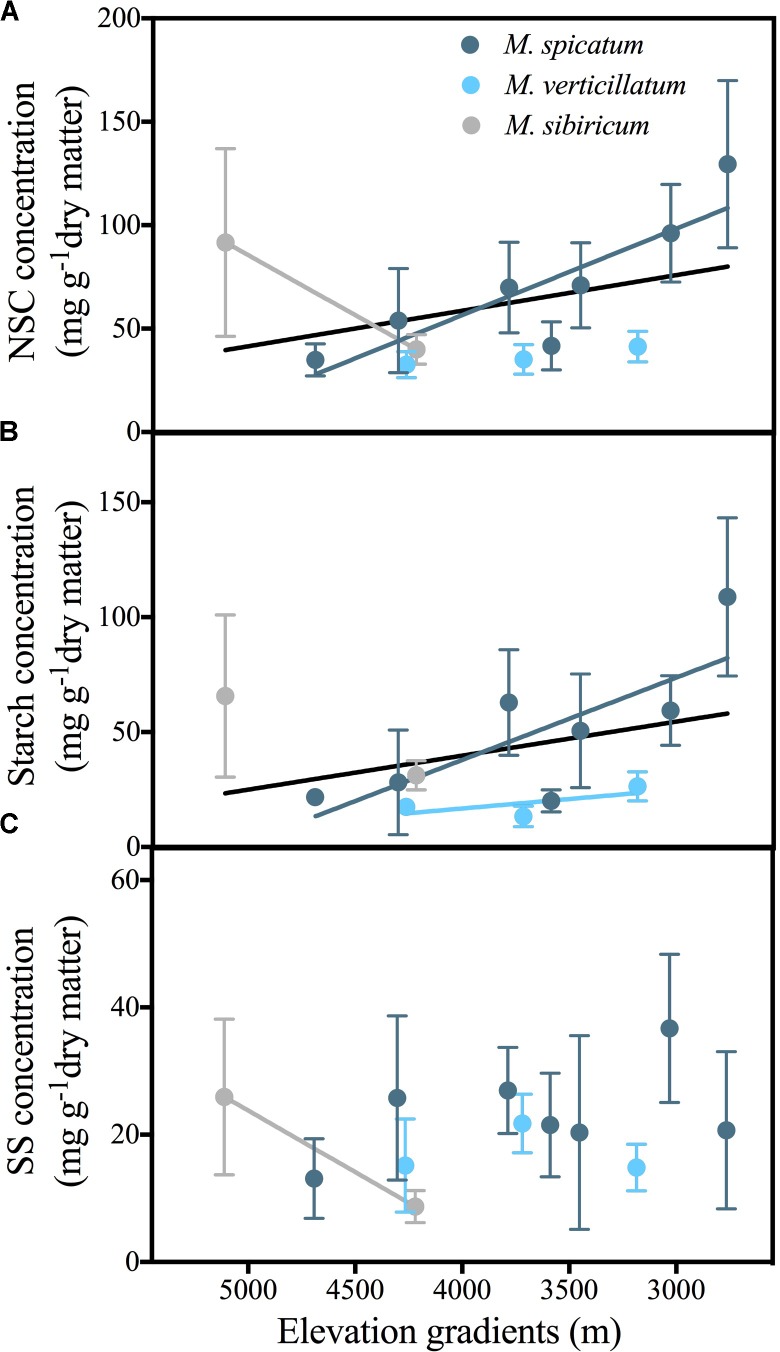
Relationships of the non-structural carbohydrate (NSC) **(A)**, starch **(B)**, and SS (soluble sugars) of leaves **(C)** in response to elevation for three *Myriophyllum* species (*M. spicatum*, *M. verticillatum* and *M. sibiricum*) combined at the Qinghai-Tibetan Plateau, China. Black lines represent the combined regression lines of three *Myriophyllum* species. Only the significant relationships are presented. Please see Appendix S2 for statistical details. The data are presented with a mean ± 2 standard error. NSC = SS + starch.

Overall, across the three *Myriophyllum* species, NSC concentrations in the leaves were observed to decrease significantly with elevation (**Figure 2A**). In most sampled water bodies, starch was the primary component of leaf NSC (mean 61.7%, **Figure 2**). Leaf starch concentrations also decreased with elevation (**Figure 2B**); however, leaf SS did not vary significantly with elevation (*F* = 1.713, *P* = 0.144) (**Figure 2C**). Among individual species, leaf NSC and starch concentrations of *M. spicatum* and *M. verticillatum* decreased with elevation (except for NSC concentrations in *M. verticillatum*, **Figures 2A,B** and Supplementary Table 2). For *M. sibiricum*; however, NSC and SS concentrations increased significantly with elevation, although there were only two water bodies that contained this species (**Figures 2A,C** and Supplementary Table 2).

**FIGURE 3:**
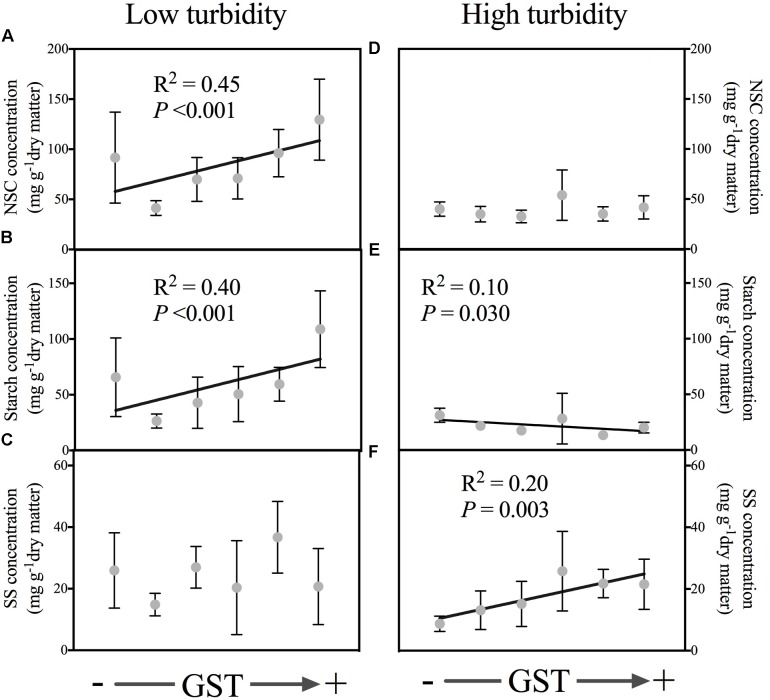
Relationships of the non-structural carbohydrate (NSC), starch and SS (soluble sugars) of leaves to mean annual temperature (GST, °C) in low turbidity (<3.97 NTU, **A–C**, including five water bodies) and high turbidity (>3.97 NTU, **D–F**, including seven water bodies) sampled water bodies, respectively. In low turbidity water bodies, the GST ranged from 4.0 to 16.3°C; In high turbidity water bodies, the GST ranged from 5.3 to 12.7°C. The data are presented with a mean ± 2 standard error. Only the significant relationships are presented. Please see **Table 1** for statistical details.

With the water bodies classified into two water turbidity groups by regression tree analysis, the leaf NSC and starch concentrations were found to decrease with higher elevations in the low turbidity group (<3.97 NTU); however, in the high turbidity group (>3.97 NTU), no such significant trend was observed (**Table 1**). In contrast, SS concentrations did not reveal a significant association with elevation, in either the low turbidity or high turbidity groups. Similar trends occurred with GST (**Table 1**). For water bodies in the low turbidity group, both NSC and starch concentrations increased significantly with GST (**Figures 3A,B**); however, the relationship between the SS concentration and GST was not significant in the high turbidity group (**Figure 3C**). Nevertheless, for water bodies with high turbidity, the starch concentrations decreased with GST, the SS concentrations increased with GST (**Figures 3D–F**). On average, plants from the low turbidity group had higher NSC and starch concentrations than those from the high turbidity group (**Figure 3**).

**Table 1:** Equations and *R^2^* and *P* values for non-structural carbohydrate (NSC), starch and soluble sugar (SS) concentrations in relation to elevation (m) and growth season temperature (GST, °C) for the *Myriophyllum* species. Model II linear equations were introduced to fit the data.

Variables	Source	Equation	*r^2^*	*P* (1-tailed)	*n*
NSC	Low turbidity	logNSC = -6.28 × 10^-5^ × elevation + 0.487	0.18	4.51 × 10^-3^	36
	High turbidity	logNSC = -9.41 × 10^-5^ × elevation + 0.581	0.01	0.32	36
Starch	Low turbidity	logStarch = -3.34 × 10^-4^ × elevation + 2.847	0.20	2.97 × 10^-3^	36
	High turbidity	logStarch = 5.21 × 10^-4^ × elevation - 0.879	0.05	0.09	36
SS	Low turbidity	sqrtSS = -1.81 × 10^-3^ × elevation + 11.118	<0.01	0.34	36
	High turbidity	sqrtSS = -3.27 × 10^-3^ × elevation + 17.538	0.09	0.04	36
NSC	Low turbidity	logNSC = 1.16 × 10^-2^ × GST + 0.129	0.45	3.62 × 10^-6^	36
	High turbidity	logNSC = 1.45 × 10^-2^ × GST + 0.070	0.01	0.30	36
Starch	Low turbidity	logStarch = 0.06 × GST + 0.943	0.40	2.00 × 10^-5^	36
	High turbidity	logStarch = -0.08 × GST + 2.145	0.10	0.03	36
SS	Low turbidity	sqrtSS = 0.33 × GST + 0.781	0.05	0.09	36
	High turbidity	sqrtSS = 0.51 × GST - 0.242	0.20	3.07 × 10^-3^	36


The GST and water turbidity comprised the two critical factors that affected the leaf NSC and starch concentrations across all elevations (Supplementary Figure 4 and Supplementary Table 3). However, the interaction between GST and turbidity was only significant in the starch concentrations (Supplementary Table 3). The NSC and starch concentrations were positively correlated with temperature, but negatively correlated with the water turbidity (Supplementary Figure 4).

## Discussion

The majority of our results found support for the CLH, rather than the GLH. Among the three *Myriophyllum* species, our data revealed that leaf NSC concentrations decreased with elevation, although NSC concentrations in *M. sibiricum* demonstrated an increased trend with elevation (albeit there were only two water bodies involved). In contrast to our results, several previous studies from tree species reported an increasing trend of plant tissue NSC concentrations with elevation (e.g., Fajardo et al., 2012; Hoch, 2015). According to the GLH, the growth of trees is initially limited by the decreased temperatures that are inherent to higher elevations, particularly in the treeline area (Körner, 1998), because cell division and expansion are more sensitive to low temperature than photosynthesis (Palacio et al., 2014). However, aquatic environments involve more complexity than terrestrial environments (Lacoul and Freedman, 2005), in that they require additional heat to alter the water temperature, in comparison to changing the air temperature. Thus, terrestrial environments have much greater daily/seasonal changes in temperature than aquatic environments; hence, terrestrial organisms (e.g., trees) must have the capacity to tolerate a wider range of temperature ranges.

Although increased temperature has been observed to enhance the growth of several types of submerged macrophytes, this phenomenon is more significant in clear and deep lakes (Rooney and Kalff, 2000). However, in shallow/highly eutrophic lakes, the increased growth of submerged macrophytes under elevated water temperatures would be restrained due to high levels of periphyton/phytoplankton shading, unless this light-limitation is somehow overcome (Cao et al., 2014; Dalinsky et al., 2014). Additionally, submerged macrophyte responses to water temperature are also species-specific. For instance, Patrick et al. (2012) suggested that water temperature elevation, as the result of climate change, extends the growing season for *M. spicatum*, but not for *M. sibiricum*. They also found strong interactions between the *Myriophyllum* species and zooplankton abundance, which indirectly influenced shading effects due to periphyton abundance under warming water conditions (Patrick et al., 2012). All of these studies suggested that, aside from water temperature, subsurface light conditions may modify the growth of submerged macrophytes.

Our results also revealed that the turbidity of each sampled water body was the most significant factor that affected leaf NSC concentrations in all sampled *Myriophyllum* species, which also altered the relationship between NSC storage in response to GST. It is known that, at least in lowland areas, light availability (primarily influenced by periphyton/phytoplankton abundance) in the water column, controls the species richness and abundance of submerged macrophytes (Rooney and Kalff, 2000; Lauridsen et al., 2015).

In high-elevation lakes, however, water transparency was associated with suspended solids, not periphyton/phytoplankton abundance, due to low nutrient concentrations (Lacoul and Freedman, 2005). Lacoul and Freedman (2005) studied 34 lakes in the Himalayan Mountains, Nepal, and found that the lakes of the high Himalayan Mountain region (from 4200 to 5600 m in elevation) possessed much higher clarity than those at lower elevations. They found that the Secchi depth of most lakes in the high mountain (from 2900 to 3600 m in elevation) was less than 2.5 m (Lacoul and Freedman, 2005). We also found that the water bodies at above 4500 m had increased transparency, although we employed a different indicator (i.e., water turbidity). In our study, the water clarity tendency was matched with the NSC variation tendency.

Several studies on aquatic plants supported our results, and revealed that high light availability had a significant effect on the accumulation of NSC in shoots, which was positively correlated with increases in biomass (e.g., Huber et al., 2012). Indeed, at high-elevations, such as in the Hymalayan region, high-transparency aqueous habitats are highly suitable for the growth of submerged macrophytes (Lacoul and Freedman, 2006b). In addition, previous *in situ* experiments revealed that the relative growth rates (RGR) in submerged macrophytes were not significantly affected by temperature; however, photosynthetic rates were positively correlated to temperature and were similar between environments with comparable light availability (e.g., Riis et al., 2012). These data highlighted that additional NSC, and no increase in RGR, may be expected under high-temperature conditions, due to the increase of photosynthesis. Furthermore, similar trends of starch concentration in both low and high turbidities were observed. Both slopes between starch concentrations and elevation/GST in low turbidity were higher than slopes from high turbidity, which supported our hypothesis that *Myriophyllum* species were carbon limited due to the photosynthesis limitation by high water turbidity. However, starch cannot be utilized directly by the plant and must be transformed to soluble sugar for utilization (Hajirezaei et al., 2003). The SS is a successful resource supply in maintaining plant growth rather than starch. Indeed, the SS concentration is related to tissue growth and may regulate hormone concentrations to modify the plant’s morphology (Huber et al., 2012; Deng et al., 2013). In our study, the increased SS concentrations in high turbidity group may reflect the growth of leaves to avoid shading in high GST condition. However, SS concentrations were in high levels in low turbidity conditions, which may serve to response to immediate environmental changes (e.g., low light or low temperature) and sustain cell growth (Huber et al., 2012). Therefore, light availability, in conjunction with water temperature, determined the distribution and growth of submerged macrophytes (at least for the *Myriophyllum* species) in high-elevation regions.

Palacio et al. (2014) argued that NSC storage in annual herbaceous *Arabidopsis* plants is different from that of trees, in that when C becomes limiting in these annual plants, it is most likely that all stores within the plant are mobilized and consumed. We support the argument that at low elevations, the NSC storage in *Myriophyllum* species is employed for growth and respiration, and the NSC is maintained at a low level during the growing season (Madsen, 1997). However, most of the data available to date, on NSC in response to elevation is derived from the treeline ecotone, which is far different from aquatic habitats (e.g., no changes in light conditions). Our study raises a number of questions (e.g., is such NSC response trend consistent in other species and larger scale?) in terms of explaining the tradeoffs between storage and growth in submerged macrophytes under high-elevation environments.

Additionally, inorganic C limited photosynthesis is much more common in aquatic, rather than terrestrial plants (Pedersen et al., 2013). In aquatic habitats, free CO_2_ constitutes only a small proportion of C resources in the water column, as the diffusion velocity of free CO_2_ comprises only 1/10000 in the water column in contrast to the ambient atmosphere (James and Larkum, 1996). Plants growing in such environments often experience low dissolved inorganic C availability conditions, as photosynthesis reduces CO_2_ concentrations to a very low level (Jones et al., 2002; Bornette and Puijalon, 2011).

All submerged macrophytes utilize dissolved CO_2_ directly, unless there is a considerable fluctuation in the pH value of the water (Schippers et al., 2004). Many submerged macrophytes have evolved alternative capacities to obtain C (i.e., bicarbonate usage) in order to acclimatize to low dissolved CO_2_ conditions (Schippers et al., 2004; Cavalli et al., 2012). In our study, although bicarbonate concentrations in water bodies were not tested (because it is very difficult to bring the water samples back to the lab), most of the pH values from the sampled water bodies exceeded 8.22 (except one pH value 7.4). At this pH value range, the quantity of dissolved CO_2_ is very low, where bicarbonate is the dominant source of C for the *Myriophyllum* species. Lacoul and Freedman (2006b) also reported that the water bicarbonate concentration is one of the major influences (e.g., temperature, lake surface area, suspended solids, bicarbonate and dissolved phosphorus) related to aquatic plant distribution and species richness in the Himalayas; however, compared with water transparency, pH value and bicarbonate concentrations only significantly influence aquatic plant distribution and species richness over larger geographical gradients. These results highlighted that the distribution of these plants in this region is likely associated with photosynthesis, which is mainly influenced by water transparency characteristics, such as water turbidity or Secchi depth.

We also compared stoichiometric data between the Qinghai-Tibetan Plateau and the middle and lower reaches of the Yangtze River, and found that the elemental C concentrations in *M. spicatum* were lower in the first region (Qinghai-Tibetan Plateau: 344.7 mg g^-1^; middle and lower reaches of the Yangtze River: 359.9 mg g^-1^) (Xing et al., 2013; Wang et al., 2015). This result at least partly reflected that there was support for carbon limitation (i.e., photosynthesis is limited by low temperature/low light condition) as an explanation in *M. spicatum*, in terms of how this species responded to higher elevations, which indicated high physiological plasticity in submerged macrophytes under different environmental conditions (Santamaría, 2002; Bornette and Puijalon, 2011).

## Conclusion

The leaf NSC concentrations in *Myriophyllum* species decreased with elevation. We suggest that this trend in NSC is attributable to the physiological plasticity of submerged macrophytes in response to light availability (e.g., water turbidity in our study). Our results implied no support for the GLH (i.e., harsh environmental conditions restricted cell division and expansion). At least two environmental factors (i.e., temperature and light availability/water turbidity) induced photosynthesis decreases might explain the NSC responses for submerged macrophytes in response to elevation at the Qinghai-Tibetan Plateau. More data is required in terms of multiple species to research how the aquatic plants responds to a wide range of elevations. The results will help the accurate prediction of plant responses to current and future climate changes worldwide.

## Author Contributions

DX and DY conceived the ideas, DX, ZWu, ZWa, and QW performed the field sampling and lab analysis, DX, ZWu, and HC analyzed the data and drafted the paper. All authors strongly contributed to writing the paper.

## Conflict of Interest Statement

The authors declare that the research was conducted in the absence of any commercial or financial relationships that could be construed as a potential conflict of interest.

## Footnotes

1 http://www.r-project.org.

## References

[B1] Adams H. D. Germino M. J. Breshears D. D. Barron-Gafford G. A. Guardiola-Claramonte M. Zou C. B. (2013). Nonstructural leaf carbohydrate dynamics of *Pinus edulis* during drought-induced tree mortality reveal role for carbon metabolism in mortality mechanism. *New Phytol.* 197 1142–1151. 10.1111/nph.12102 23311898

[B2] Aichner B. Herzschuh U. Wilkes H. (2010). Influence of aquatic macrophytes on the stable carbon isotopic signatures of sedimentary organic matter in lakes on the Tibetan Plateau. *Org. Geochem.* 41 706–718. 10.1016/j.orggeochem.2010.02.002

[B3] Alvarez-Uria P. Körner C. (2007). Low temperature limits of root growth in deciduous and evergreen temperate tree species. *Funct. Ecol.* 21 211–218. 10.1111/j.1365-2435.2007.01231.x

[B4] Bornette G. Puijalon S. (2011). Response of aquatic plants to abiotic factors: a review. *Aquat. Sci.* 73 1–14. 10.1007/s00027-010-0162-7 15356219

[B5] Cao Y. Li W. Jeppesen E. (2014). The response of two submerged macrophytes and periphyton to elevated temperatures in the presence and absence of snails: a microcosm approach. *Hydrobiologia* 738 49–59. 10.1007/s10750-014-1914-5

[B6] Cavalli G. Riis T. Baattrup-Pedersen A. (2012). Bicarbonate use in three aquatic plants. *Aquat. Bot.* 98 57–60. 10.1016/j.aquabot.2011.12.007

[B7] Cook C. D. K. (1990). *Aquatic Plant Book.* Amsterdam: SPB Academic Publishing.

[B8] Dalinsky S. A. Lolya L. M. Maguder J. L. Pierce J. L. B. Kelting D. L. Laxson C. L. (2014). Comparing the effects of aquatic stressors on model temperate freshwater aquatic communities. *Water Air Soil Pollut.* 225:2007 10.1007/s11270-014-2007-9

[B9] De’ath G. Fabricius K. E. (2000). Classification and regression trees: a powerful yet simple technique for ecological data analysis. *Ecology* 81 3178–3192. 10.1890/0012-9658(2000)081[3178:CARTAP]2.0.CO;2

[B10] Deng Z. M. Chen X. S. Xie Y. H. Li X. Pan Y. Li F. (2013). Effects of size and vertical distribution of buds on sprouting and plant growth of the clonal emergent macrophyte *Miscanthus sacchariflorus* (Poaceae). *Aquat. Bot.* 104 121–126. 10.1016/j.aquabot.2012.08.004

[B11] Fajardo A. Piper F. I. (2014). An experimental approach to explain the southern Andes elevational treeline. *Am. J. Bot.* 101 788–795. 10.3732/ajb.1400166 24812110

[B12] Fajardo A. Piper F. I. Cavieres L. A. (2011). Distinguishing local from global climate influences in the variation of carbon status with altitude in a tree line species. *Glob. Ecol. Biogeogr.* 20 307–318. 10.1111/j.1466-8238.2010.00598.x

[B13] Fajardo A. Piper F. I. Pfund L. Körner C. Hoch G. (2012). Variation of mobile carbon reserves in trees at the alpine treeline ecotone is under environmental control. *New Phytol.* 195 794–802. 10.1111/j.1469-8137.2012.04214.x 22765223

[B14] Fang J. Piao S. Tang Z. Peng C. Ji W. (2001). Interannual variability in net primary production and precipitation. *Science* 293 317–326. 10.1126/science.293.5536.1723a 11546840

[B15] Ganie A. H. Reshi Z. A. Wafai B. A. Puijalon S. (2014). Phenotypic plasticity: cause of the successful spread of the genus *Potamogeton* in the Kashmir Himalaya. *Aquat. Bot.* 120 283–289. 10.1016/j.aquabot.2014.09.007

[B16] Hajirezaei M. R. Börnke F. Peisker M. Takahata Y. Lerchl J. Kirakosyan A. (2003). Decreased sucrose content triggers starch breakdown and respiration in stored potato tubers (*Solanum tuberosum*). *J. Exp. Bot.* 54 477–488. 10.1093/jxb/erg040 12508058

[B17] Hoch G. (2015). “Carbon reserves as indicators for carbon limitation in trees,” in *Progress in Botany*, eds Lüttge U. Beyschlag W. (Heidelberg: Springer International Publishing), 321–346.

[B18] Hoch G. Körner C. (2009). Growth and carbon relations of tree line forming conifers at constant vs. variable low temperatures. *J. Ecol.* 97 57–66. 10.1111/j.1365-2745.2008.01447.x

[B19] Hoch G. Popp M. Körner C. (2002). Altitudinal increase of mobile carbon pools in *Pinus cembra* suggests sink limitation of growth at the Swiss treeline. *Oikos* 98 361–374. 10.1034/j.1600-0706.2002.980301.x

[B20] Huber H. Chen X. Hendriks M. Keijsers D. Voesenek L. A. Pierik R. (2012). Plasticity as a plastic response: how submergence-induced leaf elongation in *Rumex palustris* depends on light and nutrient availability in its early life stage. *New Phytol.* 194 572–582. 10.1111/j.1469-8137.2012.04075.x 22335539

[B21] Jackbsen D. (2008). “Tropical high-altitude streams,” in *Tropical Stream Ecology*, ed. Dudgeon D. (Cambridge: Academic Press), 219–256. 10.1016/B978-012088449-0.50010-8

[B22] James P. L. Larkum A. W. D. (1996). Photosynthetic inorganic carbon acquisition of *Posidonia australis*. *Aquat. Bot.* 55 149–157. 10.1016/S0304-3770(96)01074-1

[B23] Jones J. I. Li W. Maberly S. C. (2003). Area, altitude and aquatic plant diversity. *Ecography* 26 411–420. 10.1034/j.1600-0587.2003.03554.x

[B24] Jones J. I. Young J. O. Eaton J. W. Moss B. (2002). The influence of nutrient loading, dissolved inorganic carbon and higher trophic levels on the interaction between submerged plants and periphyton. *J. Ecol.* 90 12–24. 10.1046/j.0022-0477.2001.00620.x

[B25] Joshi J. Schmid B. Caldeira M. Dimitrakopoulos P. Good J. Harris R. (2001). Local adaptation enhances performance of common plant species. *Ecol. Lett.* 4 536–544. 10.1046/j.1461-0248.2001.00262.x

[B26] Klimeš L. Klimešová J. Èížková H. (1999). Carbohydrate storage in rhizomes of *Phragmites australis*: the effects of altitude and rhizome age. *Aquat. Bot.* 64 105–110. 10.1016/S0304-3770(99)00016-9

[B27] Körner C. (1998). A re-assessment of high elevation treeline positions and their explanation. *Oecologia* 115 445–459. 10.1007/s004420050540 28308263

[B28] Lacoul P. Freedman B. (2005). Physical and chemical limnology of 34 lentic waterbodies along a tropical-to-alpine altitudinal gradient in Nepal. *Int. Rev. Hydrobiol.* 90 254–276. 10.1002/iroh.200410766

[B29] Lacoul P. Freedman B. (2006a). Environmental influences on aquatic plants in freshwater ecosystems. *Environ. Rev.* 14 89–136. 10.1139/a06-001

[B30] Lacoul P. Freedman B. (2006b). Relationships between aquatic plants and environmental factors along a steep Himalayan altitudinal gradient. *Aquat. Bot.* 84 3–16. 10.1016/j.aquabot.2005.06.011

[B31] Lauridsen T. L. Jeppesen E. Declerck S. A. J. De Meester L. Conde-Porcuna J. M. Rommens W. (2015). The importance of environmental variables for submerged macrophyte community assemblage and coverage in shallow lakes: differences between northern and southern Europe. *Hydrobiologia* 744 49–61. 10.1007/s10750-014-2055-6

[B32] Loayza-Muro R. Marticorena-Ruíz J. K. Palomino E. J. Merritt C. Breeuwer J. A. J. Kuperus P. (2013). Ultraviolet-B-driven pigmentation and genetic diversity of benthic macroinvertebrates from high-altitude Andean streams. *Freshw. Biol.* 58 1710–1719. 10.1111/fwb.12161

[B33] Madsen J. D. (1997). Seasonal biomass and carbohydrate allocation in a southern population of Eurasian watermilfoil. *J. Aquat. Plant Manag.* 35 15–21. 10.21236/ADA327968

[B34] Madsen T. V. Brix H. (1997). Growth, photosynthesis and acclimation by two submerged macrophytes in relation to temperature. *Oecologia* 110 320–327. 10.1007/s004420050165 28307220

[B35] Olesen B. Madsen T. V. (2000). Growth and physiological acclimation to temperature and inorganic carbon availability by two submerged aquatic macrophytes species, *Callitriche cophocarpa* and *Elodea canadensis*. *Funct. Ecol.* 14 252–260. 10.1046/j.1365-2435.2000.00412.x

[B36] Palacio S. Hoch G. Sala A. Körner C. Millard P. (2014). Does carbon storage limit tree growth? *New Phytol.* 201 1096–1100. 10.1111/nph.12602 24172023

[B37] Patrick D. A. Boudreau N. Bozic Z. Carpenter G. S. Langdon D. M. Lemay S. R. (2012). Effects of climate change on late-season growth and survival of native and non-native species of watermilfoil (*Myriophyllum* spp.): implications for invasive potential and ecosystem change. *Aquat. Bot.* 103 83–88. 10.1016/j.aquabot.2012.06.008

[B38] Pedersen O. Colmer T. D. Sand-Jensen K. (2013). Underwater photosynthesis of submerged plants-recent advances and methods. *Front. Plant Sci.* 4:140. 10.3389/fpls.2013.00140 23734154 PMC3659369

[B39] Potvin C. (1986). Biomass allocation and phenological differences among southern and northern populations of the C4 grass *Echinochloa crus-galli*. *J. Ecol.* 74 915–923. 10.2307/2260223

[B40] Puijalon S. Piola F. Bornette G. (2008). Abiotic stresses increase plant regeneration ability. *Evol. Ecol.* 22 493–506. 10.1007/s10682-007-9177-5

[B41] Reich P. B. Oleksyn J. (2004). Global patterns of plant leaf N and P in relation to temperature and latitude. *Proc. Natl. Acad. Sci. U.S.A.* 101 11001–11006. 10.1073/pnas.0403588101 15213326 PMC503733

[B42] Riis T. Olesen B. Clayton J. S. Lambertini C. Brix H. Sorrell B. K. (2012). Growth and morphology in relation to temperature and light availability during the establishment of three invasive aquatic plant species. *Aquat. Bot.* 102 56–64. 10.1016/j.aquabot.2012.05.002

[B43] Rooney N. Kalff J. (2000). Inter-annual variation in submerged macrophyte community biomass and distribution: the influence of temperature and lake morphometry. *Aquat. Bot.* 68 321–335. 10.1016/S0304-3770(00)00126-1

[B44] Root E. Cantonati M. Füreder L. Pfister P. (2006). Benthic algae in high altitude streams of the Alps-a neglected component of the aquatic biota. *Hydrobiologia* 562 195–216. 10.1007/s10750-005-1811-z

[B45] Santamaría L. (2002). Why are most aquatic plants widely distributed? Dispersal, clonal growth and small-scale heterogeneity in a stressful environment. *Acta Oecol.* 23 137–154. 10.1016/S1146-609X(02)01146-3

[B46] Schippers P. Vermaat J. E. De Klein J. Mooij W. M. (2004). The effect of atmospheric carbon dioxide elevation on plant growth in freshwater ecosystems. *Ecosystems* 7 63–74. 10.1007/s10021-003-0195-z

[B47] Shi P. Körner C. Hoch G. (2008). A test of the growth-limitation theory for alpine tree line formation in evergreen and deciduous taxa of the eastern Himalayas. *Funct. Ecol.* 22 213–220. 10.1111/j.1365-2435.2007.01370.x

[B48] Wang Z. Xia C. Yu D. Wu Z. (2015). Low-temperature induced leaf elements accumulation in aquatic macrophytes across Tibetan Plateau. *Ecol. Eng.* 75 1–8. 10.1016/j.ecoleng.2014.11.015

[B49] Wiley E. Helliker B. (2012). A re-evaluation of carbon storage in trees lends greater support for carbon limitation to growth. *New Phytol.* 195 285–289. 10.1111/j.1469-8137.2012.04180.x 22568553

[B50] Wu Z. Yu D. Li X. Xu X. (2016). Influence of geography and environment on patterns of genetic differentiation in a widespread submerged macrophyte, Eurasian watermilfoil (*Myriophyllum spicatum* L., Haloragaceae). *Ecol. Evol.* 6 460–468. 10.1002/ece3.1882 26843930 PMC4729246

[B51] Xing W. Wu H. Hao B. Liu G. (2013). Stoichiometric characteristics and responses of submerged macrophytes to eutrophication in lakes along the middle and lower reaches of the Yangtze River. *Ecol. Eng.* 54 16–21. 10.1016/j.ecoleng.2013.01.026

