# Supplementary material for: Carbon Gain Limitation Is the Primary Mechanism for the Elevational Distribution Limit of Myriophyllum in the High-Altitude Plateau
Source: Front Plant Sci. 2018 Aug 2;9:1129. doi: 10.3389/fpls.2018.01129 (PMC6083828; doi:10.3389/fpls.2018.01129)
Supplement: Supplementary file 1 [file Table_1.pdf]

Supplementary Material

Carbon gain limitation is the primary mechanism for the elevational distribution limit of *Myriophyllum* in the high-altitude plateau

Dong Xie[*], Zhigang Wu, Han Y. H. Chen, Zhong Wang, Qiang Wang, Dan Yu[*]

*** Correspondence:** Corresponding Author: xiedong0123@gmail.com; lakeyd@163.com

1 Supplementary Figures and Tables

1.1 Supplementary Figures

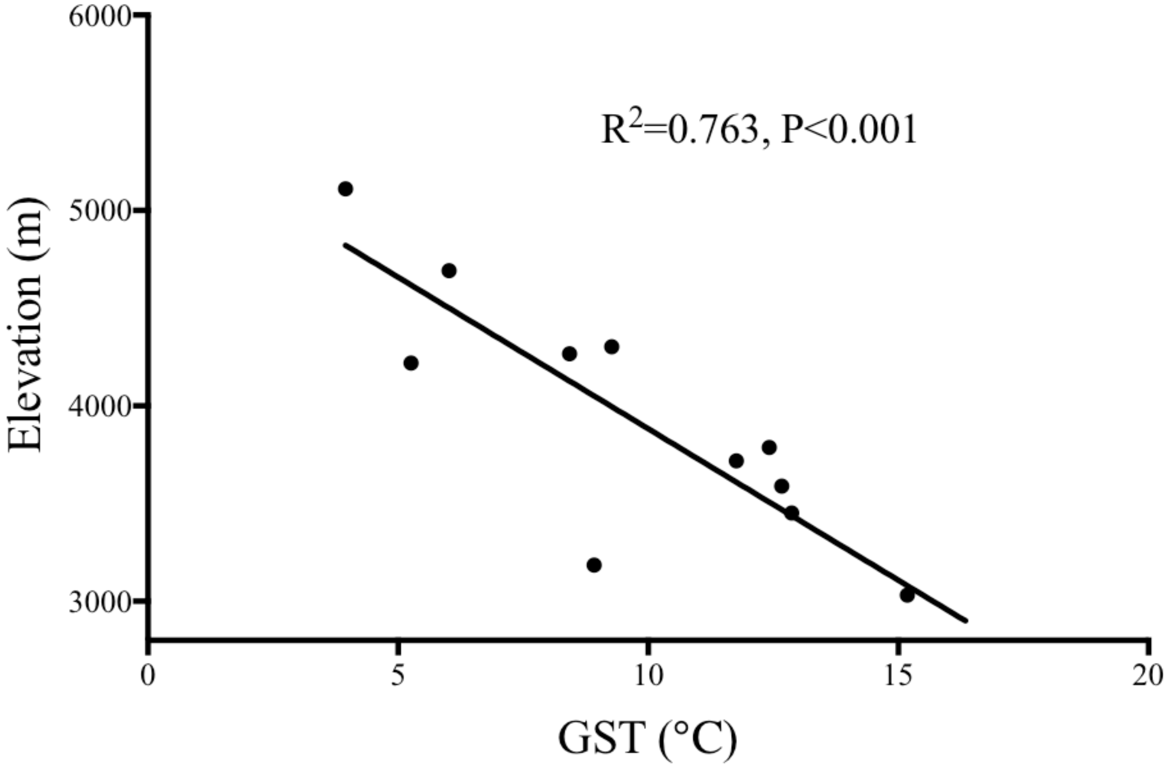

Supplementary Figure 1. Relationships of elevation and growth season temperature (GST) from the 12 sampling water bodies. *R* and *P* values of Pearson correlation are shown.

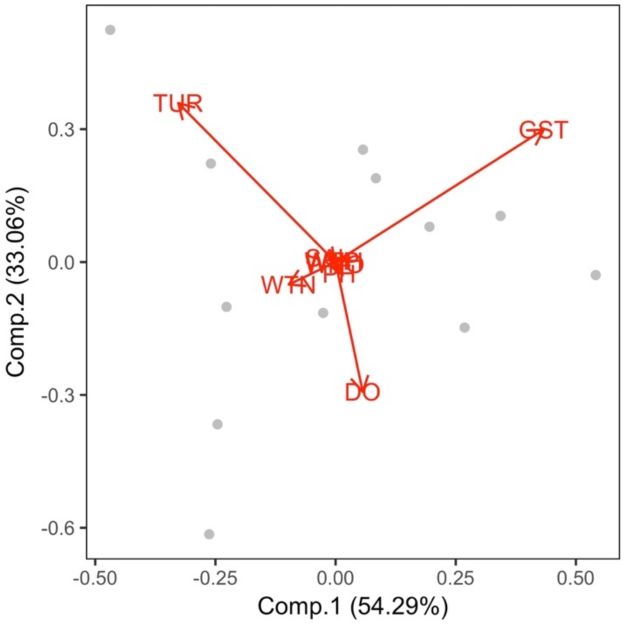

	GST	pH	salinity	DO	TUR	WTN	WTP	WNH	WNO
Comp.1	0.781	-	-	0.102	-0.590	-0.176	-	-	-
Comp.2	0.539	-	-	-0.528	0.648	-	-	-	-

Supplementary Figure 2. Correlation between the first two components of the PCA and the 9 variables (growth season temperature (GST), pH, salinity, dissolved oxygen (DO), turbidity(TUR), water total nitrogen (WTN), water total phosphorus (WTP), NH_4^+(WNH) and NO_3^-(WNO)). Loadings for the components are shown.

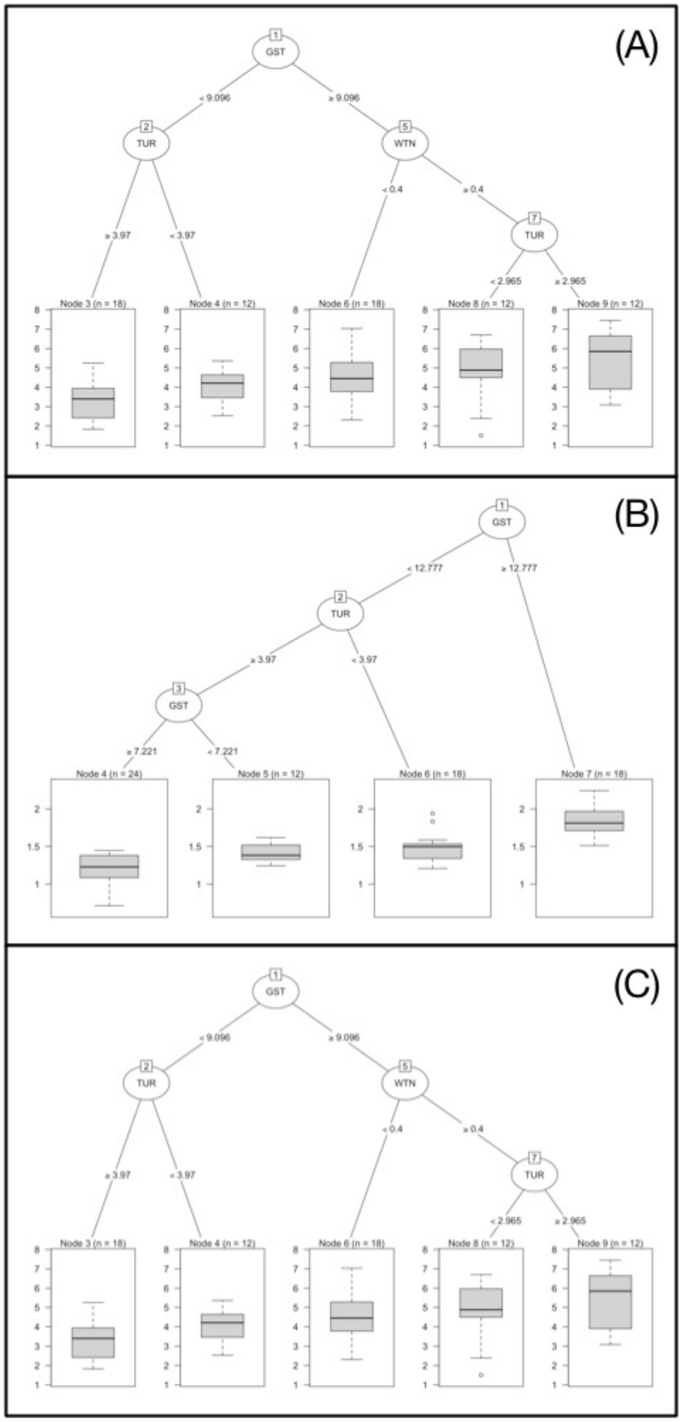

Supplementary Figure 3. Regression tree showing factors that drive NSC (A), starch (B) and SS (C) in the Qinghai-Tibetan Plateau. Length of the nodes is proportional to the reduction in the overall deviance. The explanatory variables included are growth season temperature (GST), dissolved oxygen, turbidity (TUR) and total nitrogen (WTN) derived as linear combinations of variables that explain 87.35% of the variance of the environment data.

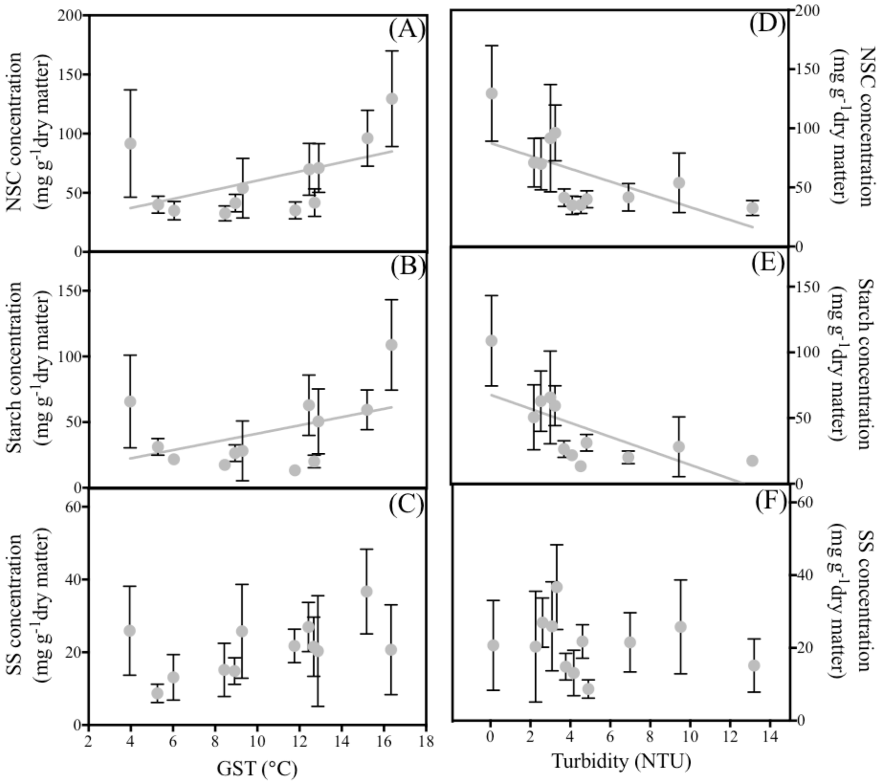

Supplementary Figure 4. Relationships of the leaf non-structural carbohydrate (NSC) (A and D), starch (B and E) and SS (soluble sugars) (C and F) to growth season temperature (GST) and water turbidity of the all three *Myriophyllum* species from 12 sampling water bodies. The data are presented as the mean ± 2 standard errors. NSC = SS + starch

1.2 Supplementary Tables

Supplementary Table 1 Principal characteristics of the 12 sampling water bodies from Qinghai-Tibetan Plateau, China. For water physical and chemical characteristics, the mean values were given (n = 3). All samples were collected from 10:00 to 15:00. Cond: conductivity; GST = growth season temperature; Cond = conductivity; TDS = total dissolved solids; DO = dissolved oxygen; TN = total nitrogen; TP = total phosphor

No.	Species	Habitat type	Latitude(N)	Longitude(E)	Elevation (m)	GST (°C)	pH	Salinity (‰)	Cond (μS cm^{-1})	TDS (mg l^{-1})	Turbidity (NTU)	DO (mg l^{-1})	TN (mg l^{-1})	NH_4-N (mg l^{-1})	NO_3-N (mg l^{-1})	TP (mg l^{-1})
1	*M. spicatum*	River	29°54′42.41″	95°38′19.07″	2766	16.34	8.70	0.13	249	178.1	0.14	6.36	0.1	0.07	0.08	0.07
2	*M. spicatum*	Wetland	29°37′51.71″	94°22′57.11″	3031	15.18	9.17	0.05	94.2	70.9	3.32	6.07	0.5	0.05	0.10	0.23
3	*M. spicatum*	Pond	29°53′33.65″	93°27′52.02″	3452	12.87	9.47	0.06	121.3	83.9	2.26	7.74	1.0	0.18	0.07	0.54
4	*M. spicatum*	Wetland	29°18′29.23″	91°04′35.69″	3589	12.67	8.66	0.15	300.2	201.5	6.99	4.92	0.1	0.05	0.04	0.11
5	*M. spicatum*	River	29°19′28.10″	89°24′36.72″	3787	12.42	8.91	0.14	283.6	196.4	2.61	2.85	1.0	0.07	0.50	0.28
6	*M. spicatum*	Wetland	29°12′35.35″	87°24′59.83″	4303	9.27	9.57	1.10	1706	1391	9.52	4.75	2.5	0.12	0.49	0.42
7	*M. spicatum*	Wetland	30°45′15.01″	88°47′14.17″	4692	6.02	8.42	0.20	259.1	268.5	4.17	2.03	1.8	1.36	<0.01	0.14
8	*M. verticillatum*	River	37°36′32.83″	101°19′11.28″	3185	8.92	7.40	0.33	621	442.0	3.77	5.03	1.0	0.15	0.07	0.20
9	*M. verticillatum*	Wetland	29°42′03.49″	91°25′46.74″	3719	11.76	8.81	0.10	169.6	134.6	4.61	2.40	0.3	0.14	0.13	0.29
10	*M. verticillatum*	Wetland	30°33′46.69″	91°10′48.18″	4266	8.43	8.22	0.22	354.6	289.9	13.2	2.05	2.2	0.16	<0.01	0.47
11	*M. sibiricum*	Lake	34°49′39.29″	98°07′47.17″	4219	5.26	9.23	0.26	427.6	352.3	4.90	7.93	1.0	0.07	<0.01	0.26
12	*M. sibiricum*	Lake	29°41′10.86″	85°43′20.17″	5111	3.95	9.39	0.11	174.6	148.8	3.08	9.24	3.5	0.02	0.43	0.32

Supplementary Table 2 Analysis of variance of non-structural carbohydrate (NSC), starch and soluble sugar (SS) concentrations of three *Myriophyllum* species (*M. spicatum*, *M. verticillatum* and *M. sibiricum*) in response to elevational gradients. Significant P-values (<0.05) are given in bold. All data were transformed using log(x) or sqrt(x) functions.

	Source of variance	*d.f.*	Sum of squares	Mean squares	*F*	*P*
**NSC**						
M. spicatum	Elevation	6	0.4808	0.0801	6.905	**<0.001**
	Residuals	35	0.4062	116		
M. verticillatum	Elevation	2	0.0237	0.0118	1.461	0.263
	Residuals	15	0.1216	0.0081		
M. sibiricum	Elevation	1	0.0796	0.0796	5.279	**0.044**
	Residuals	10	0.1508	0.0151		
**Starch**						
M. spicatum	Elevation	6	2.628	0.4379	5.787	**<0.001**
	Residuals	35	2.649	0.0757		
M. verticillatum	Elevation	2	0.2884	0.1442	6.858	**0.0077**
	Residuals	15	0.3155	0.021		
M. sibiricum	Elevation	1	0.2068	0.2068	4.672	0.056
	Residuals	10	0.4427	0.0443		
**SS***						
M. spicatum	Elevation	6	2.323	0.3872	1.824	0.123
	Residuals	35	7.428	0.2122		
M. verticillatum	Elevation	2	0.2926	0.1463	2.094	0.158
	Residuals	15	1.0478	0.0699		
M. sibiricum	Elevation	1	1.167	1.167	8.371	**0.016**
	Residuals	10	1.394	0.1394		

* Transformed using sqrt(x) function

Supplementary Table 3 Analysis of variance of non-structural carbohydrate (NSC), starch and soluble sugar (SS) concentrations of three *Myriophyllum* species (*M. spicatum*, *M. verticillatum* and *M. sibiricum*) in response to growth season temperature (GST) and water turbidity. Significant *P*-values (<0.05) are given in bold. All data were transformed using log(x) or sqrt(x) functions.

	**Source of variance**	***d.f.***	**Sum of squares**	**Mean squares**	***F***	***P***
**NSC**	GST	1	0.0117	0.0117	16.780	**0.003**
	Turbidity	1	0.0058	0.0058	8.408	**0.020**
	GST × Turbidity	1	0.0017	0.0017	2.461	0.155
	Residuals	8	0.0056	0.0015		
**Starch**	GST	1	0.1956	0.1956	8.370	**0.020**
	Turbidity	1	0.2601	0.2601	11.129	**0.010**
	GST × Turbidity	1	0.1376	0.1376	5.886	**0.041**
	Residuals	8	0.1869	0.0234		
**SS***	GST	1	3.0296	3.0296	8.051	**0.022**
	Turbidity	1	0.0055	0.0055	0.015	0.907
	GST × Turbidity	1	1.0113	1.0113	2.693	0.139
	Residuals	8	3.0103	1.5620		

* Transformed using sqrt(x) function
